# Biped Robots Control in Gusty Environments with Adaptive Exploration Based DDPG

**DOI:** 10.3390/biomimetics9060346

**Published:** 2024-06-08

**Authors:** Yilin Zhang, Huimin Sun, Honglin Sun, Yuan Huang, Kenji Hashimoto

**Affiliations:** Graduate School of Information, Production and Systems, Waseda University, Kitakyushu 808-0135, Japan; zhangyilin@moegi.waseda.jp (Y.Z.); hannah_sun@akane.waseda.jp (H.S.); hsun@akane.waseda.jp (H.S.); gakki@toki.waseda.jp (Y.H.)

**Keywords:** biomimetics exploration, reinforcement learning, biped robot, wind disturbance, adaptive exploration

## Abstract

As technology rapidly evolves, the application of bipedal robots in various environments has widely expanded. These robots, compared to their wheeled counterparts, exhibit a greater degree of freedom and a higher complexity in control, making the challenge of maintaining balance and stability under changing wind speeds particularly intricate. Overcoming this challenge is critical as it enables bipedal robots to sustain more stable gaits during outdoor tasks, thereby increasing safety and enhancing operational efficiency in outdoor settings. To transcend the constraints of existing methodologies, this research introduces an adaptive bio-inspired exploration framework for bipedal robots facing wind disturbances, which is based on the Deep Deterministic Policy Gradient (DDPG) approach. This framework allows the robots to perceive their bodily states through wind force inputs and adaptively modify their exploration coefficients. Additionally, to address the convergence challenges posed by sparse rewards, this study incorporates Hindsight Experience Replay (HER) and a reward-reshaping strategy to provide safer and more effective training guidance for the agents. Simulation outcomes reveal that robots utilizing this advanced method can more swiftly explore behaviors that contribute to stability in complex conditions, and demonstrate improvements in training speed and walking distance over traditional DDPG algorithms.

## 1. Introduction

In recent years, the field of bipedal walking robots has witnessed a series of groundbreaking achievements in robot technology [1]. According to a review by Mikolajczyk and colleagues, new research focuses on the integration and optimization of gait improvement, drive systems, sensors, and control systems [2]. Initially, the development and testing of these robots were primarily concentrated in highly controlled laboratory settings. However, as technology matured and application areas broadened, these robots are now extensively used in outdoor exploration and complex disaster relief missions [3,4], demonstrating the vast potential of robotic technology in practical operations, as noted by Bogue [5]. Furthermore, these robots have taken on important roles as navigators in busy urban areas. Cesar-Tondreau and other researchers are studying ways to improve autonomous robot navigation using imitation learning [6].

However, as bipedal robots are increasingly deployed in various complex environments, the natural challenges they face also multiply, and the complexity of these challenges grows in direct proportion to the scope of their applications. According to research by Xie et al., maintaining the stability and walking efficiency of robots in diverse environments necessitates addressing a multitude of technical challenges [7]. Among the many challenges, one of the most common for bipedal robots is dealing with the impact of wind [8]. Wind, particularly notable in outdoor environments, poses significant difficulties in maintaining robot stability and walking efficiency due to the unpredictability of its strength and direction, more so than other environmental disturbances such as rain, snow, and terrain changes [9]. To ensure stability in such conditions, researchers are exploring methods to enhance the robots’ sensory capabilities, such as installing advanced sensors to detect and predict changes in wind speed and direction and optimizing control algorithms to enable real-time adjustments in the robots’ gait and body posture to cope with sudden wind changes. This aspect of the research has been elaborately discussed by Ficht and Behnke, who explored the latest developments in related hardware design [10].

In the current landscape of bipedal robot control strategies facing the challenge of walking in windy conditions, approaches can be broadly categorized into model-based and data-driven methods [11]. Model-based strategies leverage a deep understanding of the robot’s physical characteristics to construct precise physical models for controlling the robot’s movements [12]. For instance, Qin and colleagues significantly enhanced stability in windy conditions by optimizing the design of regression models to adjust wind angles, walking speed, and pressing force. A notable advantage of this method is its interpretability and higher safety performance [13]. As described in [2], model-based approaches require fewer computational resources, which facilitates easier deployment while exhibiting greater stability and robustness, especially in handling noise. However, despite their theoretical advantages, model-based methods might struggle in practical applications due to the specificity of environments and terrains [14]. In the field of adaptive control, some researchers have proposed adaptive control methods for robot control. For example, ref. [15] proposed an adaptive controller based on the reparameterization of the Jacobian matrix. This controller successfully achieved precise trajectory tracking on parallel robots with kinematic and dynamic uncertainties and demonstrated its effectiveness through simulations and experiments in complex application scenarios, such as 2-DOF RPR and 3-DOF redundantly driven cable robots. However, adaptive control is generally suitable for linear or simply nonlinear systems. Although adaptive control has an online adjustment mechanism, the adjustment speed and range are usually limited. For wind, its highly nonlinear characteristics make it difficult for the control system to accurately capture its dynamic properties. In contrast, reinforcement learning-based control methods can better adapt to complex nonlinear systems and maintain adaptability to various terrains and environments.

In contrast, data-driven methods optimize through trial and error, using vast amounts of environmental data to enhance adaptability to different settings. These approaches often involve machine learning techniques that extract patterns from historical behavioral data and predict optimal future actions. This is discussed in [16], which illustrates that data-driven methods effectively adapt to complex and variable environmental conditions.

Reinforcement learning-based strategies for bipedal robot gait control aim to enable robots to autonomously learn and optimize their walking methods to suit varied ground and environmental conditions. To enhance the adaptability of these learning strategies while reducing their computational demands, current research trends towards integrating reinforcement learning with conventional control methods [17]. For example, Li and colleagues developed a hierarchical framework for quadruped robots’ gait planning that combines the DDPG algorithm with Model Predictive Control (MPC), achieving optimal action control [18]. Moreover, since the introduction of the soft actor-critic (SAC) algorithm, reinforcement learning-based gait control methods have made significant strides in the field of robotics [19].

In reinforcement learning-based control methods such as DDPG, agents adjust their actions based on rewards provided by the environment to achieve higher rewards. During the learning process, agents must perform many trial-and-error operations. This includes adding noise to the actions from the Actor model to make exploration more random [20,21]. This random exploration strategy aids the agent in investigating a wider array of potential solutions. However, this trial-and-error learning method also faces challenges, particularly when environmental rewards are sparse. In situations with sparse rewards, agents may lack sufficient incentive signals to quickly learn and optimize their behavioral strategies, leading to slower convergence rates in the learning process [22]. Combining HER [23] with adaptive exploration can make the learning process more stable and efficient. This approach provides a direct way to control and helps address the issue of sparse rewards in reinforcement learning.

Previous studies have often used Gaussian noise to simulate the uncertainty of natural wind, such as the study by Zheng et al., which described the stochastic time-varying characteristics of natural wind using sinusoidal Gaussian noise [24]. However, natural wind is not entirely random but often fluctuates around a mean state, with current and previous wind speeds exhibiting temporal correlations. The Ornstein–Uhlenbeck (OU) process naturally reverts to a long-term mean and, by adjusting the parameters of the OU process, the correlation length of the process values can be controlled, allowing the model to adjust based on actual data to reflect short or long memory effects.

Our research, inspired by how human children learn to walk in unstable environments, focuses on addressing the issue of insufficient exploration under the influence of wind in the walking control of bipedal robots. The main contributions of this paper can be specifically summarized as follows:

1. Introduction of an Adaptive Bio-Inspired Exploration Mechanism for DDPG: By incorporating an environment state-based exploration parameter adjustment module, the algorithm increases exploration when wind speeds are high, thereby enhancing learning efficiency.
2. Optimization Using Hindsight Experience Replay: This method allows the agent to learn from what might initially be considered useless failed experiences when it fails to achieve the final goal, thus accelerating the learning process.
3. Reconstruction of the Reward Function: This is optimized to address issues of sparse rewards and excessive changes in robot joint acceleration, speeding up model training and reducing joint torques. The function not only considers the basic need for the robot to maintain an upright posture but also emphasizes its ability to maintain dynamic balance and effective walking under the influence of wind.
4. Simulation of Wind Speed Using the OU Process: ref. [25] is used to generate wind speed data with temporal correlations, enhancing the reliability of the robot’s real-world walking performance.

## 2. Materials and Methods

In this section, we will first outline the fundamental principles of reinforcement learning and delve into the analysis of the proposed model. Subsequently, we will discuss the optimization methods of the model.

### 2.1. Principles of Reinforcement Learning

**Figure 1:**
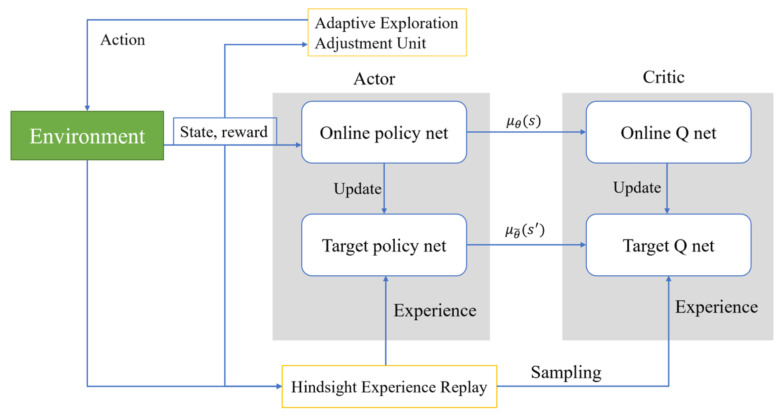
Comprehensive overview of the proposed model.

The fundamental principle of reinforcement learning is that an agent determines its actions through interaction with the environment, based on the state of the environment, and by receiving rewards or punishment signals as feedback [26]. The agent’s task is to learn, through trial and error, which actions to take to maximize its cumulative reward over the long term. A reinforcement learning model is often formalized as a Markov Decision Process (MDP) [27], where each state *S* is defined by the environment’s instantaneous configuration, and the actions *A* represent all possible operations that the agent can take in that state. Whenever the agent executes an action, the environment responds and transitions to a new state according to a probability transition function *P*, and the agent receives a reward *R*. This reward typically depends on the previous state, the chosen action, and the new state. The probability distribution of the next state in this process depends only on the current state and action, not on prior history. During the learning process, the agent tries to discover a policy π, which is a mapping function from states to actions, to maximize the expected cumulative reward from time step *t*. The optimal policy $\pi^{*}$ is defined by:

(1) $$\pi^{*}=\arg\max_{\pi }\sum_{t=0}^{T}E_{\pi \left(s_{t},a_{t}\right)∼\rho_{\pi }}\left[\gamma^{t}r\left(s_{t},a_{t}\right)\right]$$

where $\rho_{\pi }\left(s_{t},a_{t}\right)$ is the action *a* output by policy π in state *s*. DDPG is a reinforcement learning algorithm that combines policy gradient methods with deep learning. It is primarily used for problems with continuous action spaces and is a variant of the Actor-Critic method. The adaptive structure based on DDPG proposed in this article is shown in Figure 1. The Actor in the figure contains two modules: the Online policy net and the Target policy net. The Online policy net uses the current policy to determine the actions to be taken in a given state, while the Target policy net is a slightly lagged version of the policy network used to stabilize the learning process. Its updates are periodic, not occurring at every learning iteration. Experience Replay is used to store the experience generated by the agent’s interaction with the environment (including states, actions, rewards, and the next state). Experiences are randomly drawn from this pool for training to break the temporal correlation between experiences and improve learning efficiency. The Adaptive Exploration Adjustment Unit is used to adjust the exploration strategy based on the uncertainty of the environment.

### 2.2. Ornstein–Uhlenbeck Process

The Ornstein–Uhlenbeck (OU) process is characterized as a mean-reverting model, implying that wind speed oscillates around a long-term average and gradually returns to this mean. The OU process incorporates several parameters—mean, volatility, and reversion speed—which allow the model to be flexibly adapted to specific climatic conditions or wind speed data pertinent to particular geographical locations. This process provides a framework for modeling the evolution of a stochastic variable $X_{t}$ over time, with its dynamics equation expressed as:

(2) $$dX_{t}=\theta \left(\mu -X_{t}\right)dt+\sigma dW_{t}$$

**Figure 2:**
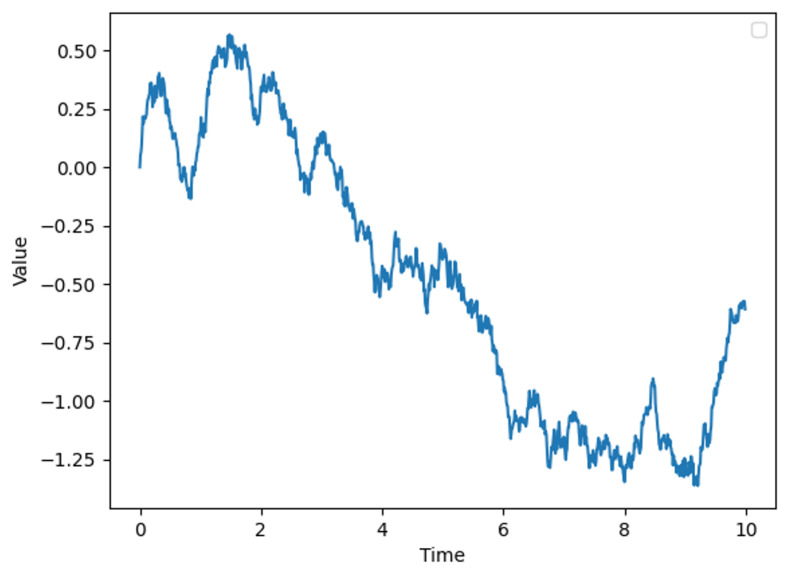
Ornstein–Uhlenbeck process.

Here, $X_{t}$ denotes the state variable at time *t*, such as wind speed. The parameter θ is the rate parameter that describes the speed at which the variable reverts to the mean μ, which represents the long-term average state of the wind speed. σ characterizes the volatility of the process, reflecting the natural fluctuation magnitude of the wind speed. $dW_{t}$ represents a standard Brownian motion, indicating random perturbations. By setting θ=0.15, μ=0 and σ=0.3, we obtain a time series graph of the OU process as shown in Figure 2, where the x-axis represents time and the y-axis denotes the magnitude of wind speed.

**Figure 3:**
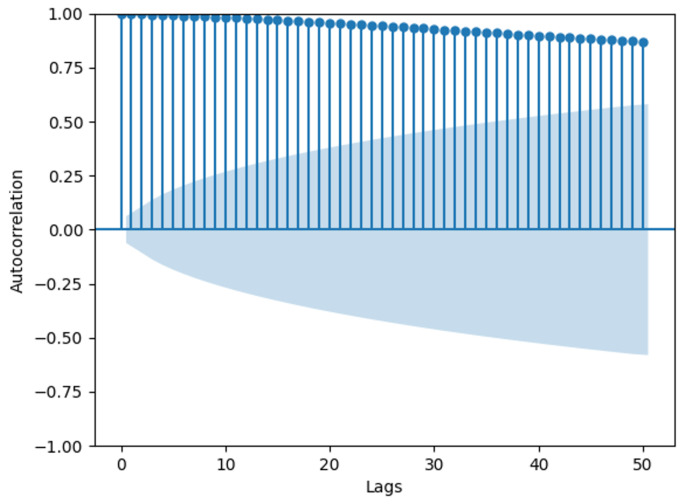
Autocorrelation of OU process.

Figure 3 shows an autocorrelation graph of the OU process, illustrating the correlation of the process with itself at different time intervals. The y-axis shows the values of the autocorrelation coefficients, ranging from −1 to 1. A value of 1 indicates perfect positive correlation, −1 indicates perfect negative correlation, and 0 implies no correlation. The x-axis shows the number of lags, where Lag 1 indicates the correlation between consecutive observations in the sequence, Lag 2 indicates the correlation between observations one time point apart, and so forth. The shaded area in the graph represents the 95% confidence interval statistically, with autocorrelation values outside this interval considered statistically significant [28]. The graph reveals significant autocorrelation within 50 time lags in the time series. In this context, if the time series represents wind speed data, the high autocorrelation values indicate that wind speed at a given time is strongly correlated with wind speeds at previous times. This temporal dependency implies that past wind speeds can be used to predict future wind speeds. Using autocorrelation in modeling wind speed has several advantages: it improves predictive accuracy by leveraging historical data for short-term forecasting, captures inherent temporal patterns in wind speed data, and most importantly, enhances simulation models by replicating the observed temporal dynamics, making the wind speed simulations more realistic. These advantages are essential for applications such as energy production forecasting and weather prediction.

In this study, an actor based on neural networks demonstrates effective learning of the nonlinear features within the wind speed sequence, thereby adapting well to such environments. This integration of autocorrelation into the model offers a comprehensive overview of the proposed model’s capability to accurately predict and adapt to varying wind speeds, ultimately leading to more realistic simulations.

### 2.3. Hindsight Experience Replay

Despite being initially designed for value-based methods, experience replay also plays an important role in policy gradient methods, including DDPG. DDPG is an Actor-Critic method that combines value function approximation and policy optimization, and the critic network is used to estimate the action-value function, while the actor network is used for policy optimization. As DDPG is an online learning algorithm, each state-action pair affects the network updates. By storing and reusing past experiences, experience replay can quickly converge the critic network, significantly reducing the number of environment interactions needed and improving training efficiency. In DDPG, the critic network is updated using the Temporal Difference (TD) target, which depends on the current policy and estimated action values. Experience replay smooths the changes in the target values, thereby enhancing the training effectiveness of the Critic network. HER is a technique designed to generate positive experiences by reevaluating failed episodes, thereby aiding in the training of agents, particularly when the data predominantly comprise unsuccessful attempts. In typical scenarios, an agent collects training data through exploration, which largely consist of failed attempts. Given the challenges associated with optimizing strategies that meet objectives, HER is employed to navigate these difficulties.

After the completion of an event, HER extracts segments of the failure trajectories and transforms them into successful outcomes. This transformation involves selecting a specific state from the extracted trajectory to serve as a new, virtual goal. Subsequently, the rewards for the trajectory from its initial state to this new goal are recalculated, assuming that reaching the virtual goal equates to a successful outcome. These recalculated rewards are stored as if the objective was achieved. These trajectories are added to the database, helping to explore new solution spaces.

In this study, the task objective is focused on robotic walking. During the attempt to walk, the robot may fall, but the steps before the fall still hold value. A fall usually occurs within 0.5 s, with each simulation time step being 0.025 s. To effectively utilize these experiences, we select the states from the 20 steps before the fall as successful targets, and assign a success reward of 10 to successful attempts, while failed attempts are also stored in the experience pool without additional rewards. This means that even though a fall eventually occurs, the robot’s position and state before the fall are redefined as virtual successful targets. Reevaluating failed trajectories and redefining their rewards as successes increases the proportion of successful experiences in the training data, and Algorithm 1 describes this process. This helps the robot learn to avoid falling in future attempts, leading to more stable and enduring walking in practical tasks.
**Algorithm 1** Modified HER Algorithm for Retaining Experience with Adjusted Rewards  1:Initialize environment and agent  2:state_history←[]  3:fallen_index←−1  4:**while** task not finished **do**  5:    action←agentselectsaction(current_state)  6:    next_state,reward,done,info←environmentexecutesaction(action)  7:    Append current_state to state_history  8:    **if** info[‘fallen’]=True **then**  9:        done←True10:      fallen_index←length(state_history)11:  **end if**12:  current_state←next_state13:**end while**14:**if** fallen_index≥20 **then**15:   successful_states←state_history[0:fallen_index−20]16:   last_actions←state_history[fallen_index−20:fallen_index]17:  Add a reward of 10 to the reward of successful_states18:  Process successful_states for further learning as successful attempts19:  Store last_actions in experience pool without additional reward20:**end if**

### 2.4. Network Architecture

In the proposed article, an Actor-Critic method is presented wherein both the actor and the critic are approximated by neural networks. The actor network is designed to approximate the policy by determining the actions to take, while the critic network evaluates the value of these actions, assessing the policy’s effectiveness.

**Figure 4:**
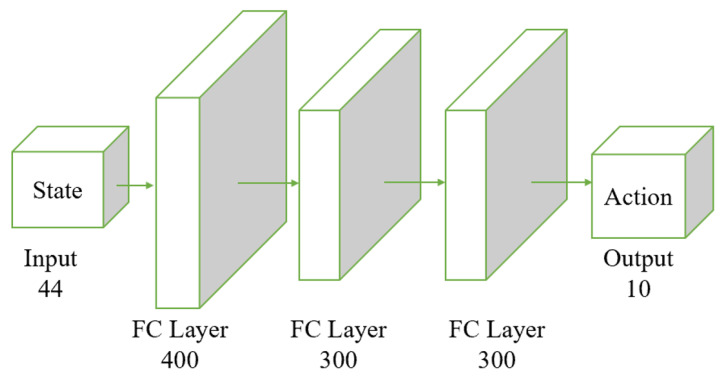
Structure of the actor network. The input layer receives a 44-dimensional state vector. The state vector passes through three fully connected layers: initially through 400 neurons, followed by two consecutive layers of 300 neurons each. The final output layer produces a 10-dimensional action vector. The fully connected layers transform the input state vector through these stages, ultimately producing the action vector as the output.

Both the actor and the critic networks are based on the Multi-Layer Perceptron (MLP) architecture, incorporating fully connected layers and activation functions. The input to the actor is the observed state, with its output being deterministic actions. The input to the critic includes both the observed state and the action output by the actor, and its output is a score representing the valuation of the actions taken by the actor, as shown in Figure 4.

The input layer receives the state with a dimensionality of 44. The data then flow through two fully connected layers; the first layer contains 400 neurons, and the second contains 300 neurons. The final output layer generates the actions; in this example, the output dimension is 10, representing 10 different action parameters.

**Figure 5:**
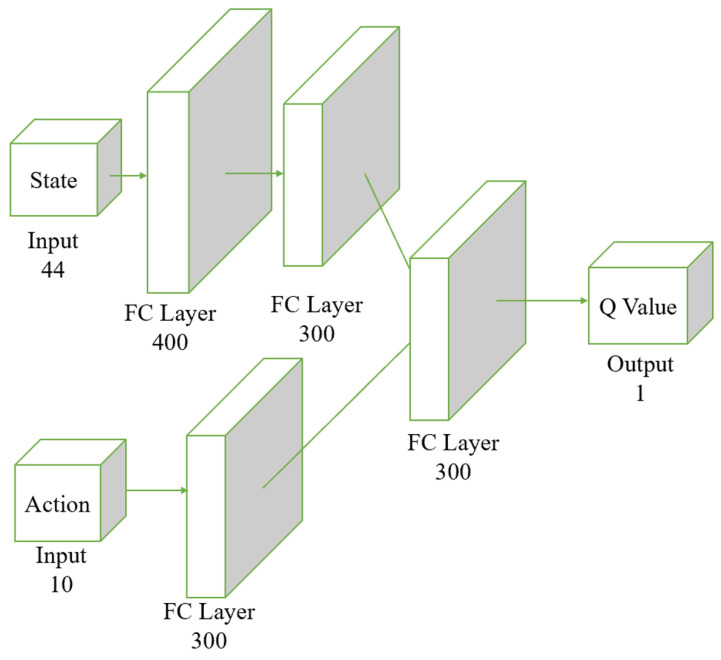
Structure of the critic network. The input layer receives a 44-dimensional state vector along with an action vector of dimension 10. The state vector passes through two fully connected layers, initially through 400 neurons, followed by 300 neurons. The action vector passes through a separate fully connected layer of 300 neurons. The processed results of both the state and the action are combined in another fully connected layer of 300 neurons, culminating in the output of the Q Value.

Figure 5 illustrates the structure of the critic network. In the Actor-Critic structure, the critic network needs to input both state and action to predict the Q value. The Q value represents the expected return of taking a specific action in a given state, so considering both inputs is crucial for accurate predictions. By using separate streams for state and action, the network can learn the features of each input type independently without interference. This separation allows the network to capture the complex dependencies between states and actions more effectively. Each stream, state and action has fully connected (FC) layers to extract the complex features relevant to each input. The state stream starts with an FC layer of 400 units followed by a 300-unit FC layer, which helps to identify essential characteristics of the environment. Similarly, the action stream, with a 300-unit FC layer, focuses on the specific details of the chosen action. After processing state and action separately, their features are combined in a subsequent FC layer. This integration step enables the network to learn the interactions between state and action, which is essential for accurately computing the Q value. This two-stream architecture improves learning efficiency by allowing parallel processing of state and action features, leading to faster convergence during training and better overall performance. The critic network with a two-stream neural network structure enhances its learning and generalization ability, leading to more accurate Q value predictions in complex environments.

### 2.5. Reward Function

In the domain of reinforcement learning tasks, the construction of a robust reward function is crucial as it furnishes the agent with objectives and guidance, crucial for its learning processes [29]. The objective of this part is to engineer a reward function that enables the robot to maintain stable locomotion even in windy environments.

#### 2.5.1. Forward Progress Reward

Given the substantial impact of wind on the robot’s velocity and directional stability, which can induce instability during movement, it is imperative not to assign excessive rewards for forward velocity to ensure stability in windy conditions. Consequently, the robot’s forward velocity $v_{x}$ is incorporated into the reward system to encourage forward movement, but it is assigned a moderated weight of 0.8. This approach aims to balance the robot’s progress with the necessity to withstand destabilizing wind effects, ensuring an optimal adaptation to challenging environmental conditions.

#### 2.5.2. Stability Reward

To enhance the robot’s capability to maintain stability in windy conditions, we specifically design the reward function to emphasize the duration of stable, upright locomotion. Recognizing the increased difficulty for the robot to remain balanced in windy versus calm conditions, the reward system assigns higher weights to stability under challenging environmental factors. The stability reward is quantitatively defined within the simulation as $40\times T_{s}/T_{f}$, where $T_{s}$ is the sampling time for the system’s sensors and controllers, and $T_{f}$ is the total duration of the simulation episode. This ratio ensures that the reward is directly proportional to the fraction of the simulation time during which the robot maintains an upright and stable posture. To directly address the challenge posed by wind, the weight assigned to this stability reward is notably high, set at 40.

#### 2.5.3. Directional Penalty

To maintain path adherence under windy conditions, the robot’s deviation from the preset trajectory is penalized. In specific terms, it is the distance between the current position of the robot and the position on the path Δy, using a penalty weight of 2, to encourage close adherence while allowing for necessary adjustments due to wind-induced navigation challenges.

#### 2.5.4. Vertical Displacement Penalty

Under strong wind conditions, it is also important to penalize vertical displacement to ensure the robot does not jump while walking. Strong winds can increase the robot’s vertical displacement as it adapts to wind-induced body tilting or instability. However, under strong wind conditions, if the robot seeks to achieve higher stability, it may lower its center of gravity appropriately. Therefore, we control that if the robot’s center of gravity decreases within a certain value, it will not be penalized. We set the robot’s vertical displacement measure to 2 cm, to gauge the robot’s ability to maintain stability in the wind, and set its weight at 30. Therefore, this part of the punishment is set as:

(3) $$\begin{array}{c} r_{v}=\left\{\begin{array}{cc} 30{\left(\Delta z\right)}^{2} & \left(\Delta z>2\right)\\ 0 & \left(\Delta z\leq 2\right) \end{array}\right. \end{array}$$

where Δz is vertical displacement and $r_{v}$ represents vertical displacement penalty.

#### 2.5.5. Joint Wear Penalty

In bipedal humanoid robot locomotion, unrestricted joint usage can lead to the robot continually walking with the same foot, significantly increasing joint wear and reducing lifespan. Additionally, this behavior degrades walking performance and increases the force required to drive the robot. Consequently, cumulative joint torque is incorporated as a penalty $\sum_{i}{\left(i\cdot u_{t-1}\right)}^{2}$ in the reward function with a weight of 0.02 to mitigate these issues. The symbol $u_{t}$ represents the torque of the corresponding joint at time *t*.

#### 2.5.6. Joint Acceleration Penalty

In the reinforcement learning framework, penalizing the acceleration of robot joints can significantly reduce mechanical wear, as this strategy encourages the algorithm to favor smoother and more gradual movements during action execution, thereby decreasing the stress and wear on joints and drive systems [30]. Furthermore, acceleration penalties also aid in avoiding dynamic instability issues caused by rapid movements, ensuring the stability of robot behavior during the execution of complex tasks, especially when operating in dynamic and unpredictable environments.

In this study, we use the integral of joint acceleration $-\sum_{i}{\left({\ddot{\theta }}_{i}\right)}^{2}$ as a penalty. Since this penalty accumulates very quickly and joint acceleration is inevitable, we have adjusted its weight to 0.03.

Overall, the reward function proposed in this paper is as follows:

(4) $$r_{t}=0.8v_{x}+30r_{v}-0.02\sum_{i}{\left(u_{t-1}^{i}\right)}^{2}-2\Delta y^{2}-0.03\sum_{i}{\left({\ddot{\theta }}_{i}\right)}^{2}+40\;\times \;T_{s}/T_{f}$$

In which $v_{x}$ represents forward velocity, $r_{v}$ represents vertical displacement penalty, $u_{t-1}^{i}$ represents torque of the i-th joint, Δy represents horizontal displacement penalty, and ${\ddot{\theta }}_{i}$ represents joint acceleration. $T_{s}$ is the sampling time for the system’s sensors and controllers, and $T_{f}$ is the total duration of the simulation episode.

### 2.6. Adaptive Exploration Method

In reinforcement learning, the exploration level is important for encouraging the algorithm to explore a wider state space. This helps in finding better strategies to handle uncertainties such as varying wind forces. As the robot walks in windy conditions, the increase in wind level can lead to instability. Therefore, under high wind conditions, it is beneficial to increment the exploration level to allow the model to find suitable strategies for coping with the gustier weather more swiftly.

For the DDPG algorithm, the exploration level is determined by the magnitude of OU noise added to the output, with its intensity governed by the parameter σ. To appropriately increase the exploration in extreme environments while maintaining agent exploration stability under normal conditions, the calculation of σ(f) is as follows:

(5) $$\sigma \left(f\right)=\sigma_{\mathrm{base}}+\alpha \cdot \max\left(0,f-f_{\mathrm{threshold}}\right)$$

Here, $\sigma_{\mathrm{base}}$ represents the base variance value used when there is no wind or the wind speed is very minimal, ensuring that there is still some level of exploration even in calm conditions. The factor α is a proportionality factor that adjusts the influence of wind speed on the variance. The variable *f* represents the current wind force, and $f_{\mathrm{threshold}}$ is a threshold force; the variance of the noise is increased only when the wind speed exceeds this threshold. In this research, α is set to 0.3, and $f_{\mathrm{threshold}}$ to 0.35 N, which are based on the magnitude of force experienced by the robot. This aspect will be detailed in Section 3 of the article.

From the discussions, it is evident that the algorithm proposed in this study builds upon the HER and incorporates an adaptive exploration mechanism within the framework of the DDPG algorithm. This sophisticated integration allows the algorithm to effectively balance exploration and exploitation, enhancing performance and efficiency in reinforcement learning tasks. The structure of the algorithm is delineated in Algorithm 2.
**Algorithm 2** DDPG with HER and Adaptive Exploration  1:Initialize online policy network $\mu_{\theta }$ with weights θ  2:Initialize target policy network $\mu_{\theta^{\prime }}$ with weights $\theta^{\prime }\leftarrow \theta$  3:Initialize online Q network $Q_{\phi }$ with weights ϕ  4:Initialize target Q network $Q_{\phi^{\prime }}$ with weights $\phi^{\prime }\leftarrow \phi$  5:Initialize experience replay pool D to capacity *N*  6:**for** episode = 1, M **do**  7:    Receive initial observation state $s_{1}$  8:    **for** t = 1, T **do**  9:       Obtain μ from the adaptive exploration adjustment unit10:     Calculate exploration noise11:     Select action $a_{t}=\mu_{\theta }\left(s_{t}\right)+\mathrm{exploration}\;\mathrm{noise}$12:     Execute action $a_{t}$ in the environment13:     Observe reward $r_{t}$ and new state $s_{t+1}$14:     Store transition $\left(s_{t},a_{t},r_{t},s_{t+1}\right)$ in D15:     Store modified transitions with alternative goals16:     Sample random mini-batch of *K* transitions $\left(s_{i},a_{i},r_{i},s_{i+1}\right)$ from D17:     Set $y_{i}=r_{i}+\gamma Q_{\phi^{\prime }}\left(s_{i+1},\mu_{\theta^{\prime }}\left(s_{i+1}\right)\right)$18:     Update ϕ by minimizing the loss: $L=\frac{1}{K}\sum_{i}{\left(y_{i}-Q_{\phi }\left(s_{i},a_{i}\right)\right)}^{2}$19:     Update θ using the sampled policy gradient:
$\nabla_{\theta }J\approx \frac{1}{K}\sum_{i}\nabla_{a}Q_{\phi }\left(s,a\right){\left|s=s_{i},a=\mu \theta \left(s_{i}\right)\nabla_{\theta }\mu_{\theta }\left(s\right)\right|}_{s_{i}}$20:     Update the target networks:
$$\begin{array}{ccc} \theta^{\prime } & \leftarrow & \tau \theta +\left(1-\tau \right)\theta^{\prime }\\ \phi^{\prime } & \leftarrow & \tau \phi +\left(1-\tau \right)\phi^{\prime } \end{array}$$21:  **end for**22:**end for**

## 3. Results

### 3.1. Simulation Environment

For this research, training the model on an Intel Core i9-11900 CPU and an RTX 3080 GPU takes about three hours per session. The WalkingRobot model from the Simscape Multibody plugin in MATLAB 2023b is utilized. This model represents a bipedal robot designed to walk along a predetermined straight line (along the y-axis) under varying wind force conditions. To facilitate mechanical computations and accelerate simulation speeds, the robot’s feet are designed as squares, with each corner of the foot equipped with a sphere of 2.5 mm radius that serves as the mechanical contact point with the ground. The parameters of the bipedal robot are detailed in Table 1.

Simultaneously, in the virtual environment, the robot is equipped with a wind force sensor and an Inertial Measurement Unit (IMU), enabling it to gather various motion and wind force data. This instrumentation allows the robot to make targeted adjustments to its movements based on the collected information. Therefore, during the training process, the observations for the model include:

1. The twist angles of the robot’s two legs along three axes (yaw, pitch, roll).;
2. The velocities of the robot’s five joints (two ankle joints, two knee joints, one hip joint);
3. The accelerations of the robot’s five joints;
4. The angular velocities of the robot’s five joints;
5. The power output of all the joint motors.

The actuation variables for the robot are the torques applied to the ankle, knee, and hip joints. During the simulation process, each segment length, Ts, is 10 s, with a control system sampling rate, Tf, of 0.025 s, indicating that each segment is composed of up to 400 steps. However, the simulation will be stopped under certain conditions, for example, if the robot falls. The conditions that trigger a cessation of the robot’s simulation in this study are as follows:

1. The robot’s ankle, knee, and hip angles exceed 60 degrees, an indicator of an extremely unstable state or a fall;
2. The height of the robot’s center of gravity falls below 0.1 m, which implies that the robot has toppled over;
3. The robot’s position deviates more than 0.5 m from the given trajectory, signifying excessive deviation and necessitating a halt to the simulation.

### 3.2. Experiments Design

To evaluate the effectiveness of the proposed approaches, the study conducts tests on several variants of the DDPG model under different configurations:

1. The standard DDPG model;
2. An enhanced DDPG model featuring Reward Reshaping, which aims to refine the reward feedback to the agent for improved learning efficiency);
3. A DDPG model augmented with Adaptive Exploration and HER, to balance exploration and exploitation and to learn from unsuccessful attempts by reframing them as successful ones in hindsight;
4. The Proposed Method: This model integrates the Adaptive Exploration and HER with the Reward Reshaping improvements within the DDPG framework.

Due to the potential for the DDPG model’s performance to degrade in the latter stages of training, the study selects the attempt with the highest reward accrued during training as the optimal scenario for further testing. The robot’s performance is then tested based on the distance it can walk along a predetermined route within a 10-s interval. This metric of distance covered in a fixed time frame serves as a quantifiable measure to assess the effectiveness of each model variation, with the premise that a more effective model will enable the robot to walk farther within the allotted time.

### 3.3. Simulation of Wind Force

The article describes the use of OU noise to simulate wind forces. The OU noise is apt for this purpose as it exhibits short-term correlations in intensity and direction—akin to natural wind patterns—while tending to revert to a mean value over the long term.

The impact of wind forces on the robot’s walking is significant and varies with wind speed. At wind speeds of Beaufort scale 3 or lower, the wind has negligible effects on the robot’s locomotion. However, at a Beaufort scale of 7 or higher, the wind speed poses a substantial challenge, making it difficult even for humans to walk normally.

To estimate the force of wind on the robot at various wind speeds, the study employs fundamental principles of fluid dynamics. The force exerted by wind on an object can be estimated using a particular equation that considers the wind’s velocity, the surface area of the object facing the wind, the air density, and a drag coefficient that characterizes how the shape of the object affects the wind resistance [31]:

(6) $$F=\frac{1}{2}\rho AC_{d}v^{2}$$

where *F* is the force of the wind on the object (Newtons, N). ρ is the air density (approximately 1.225 kg/m^3^ at standard sea level conditions). A is the frontal area of the object facing the wind (square meters, m^3^).

In this study, we begin by postulating a drag coefficient $C_{d}$ of 1.2 and an air density ρ of 1.225 kg/m^3^. The robot under investigation features a torso dimensioned at 50 mm × 80 mm × 80 mm. Utilizing these parameters, we can calculate the average wind force exerted on the robot under varying wind conditions, as detailed in Table 2.

### 3.4. Experiments Results

To conduct a detailed comparison of the robot’s mobility capabilities under different wind speeds, we systematically set the experimental conditions with wind speeds at Beaufort scale levels 3, 4, 5, and 6. The experiment utilized four different control algorithms, which have been described in detail in Section 3.2. Therefore, to comprehensively evaluate the effectiveness of each method under various wind conditions, we conducted a total of 16 independent experiments. Each experiment consisted of 5000 episodes, with no stopping conditions set during the experimental process; the experiments concluded after 5000 episodes.

Soft Actor-Critic is a deep reinforcement learning algorithm that maximizes policy entropy, considering both rewards and the diversity of actions during learning, thus improving exploration efficiency and stability. Asynchronous Advantage Actor-Critic (A3C) is a distributed reinforcement learning algorithm that uses multiple asynchronous workers to accelerate training and employs an advantage function to reduce variance and enhance policy stability. Both SAC and A3C methods will be experimented with and their results compared.

**Figure 6:**
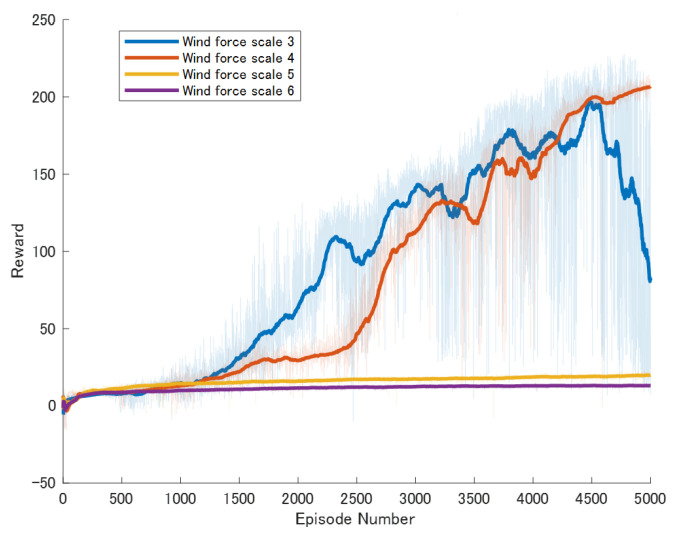
Result of The standard DDPG model. The dark lines represent the average rewards over the most recent 100 episodes for each wind force scale, while the pale areas show the rewards for each individual episode. The following figures are the same.

**Figure 7:**
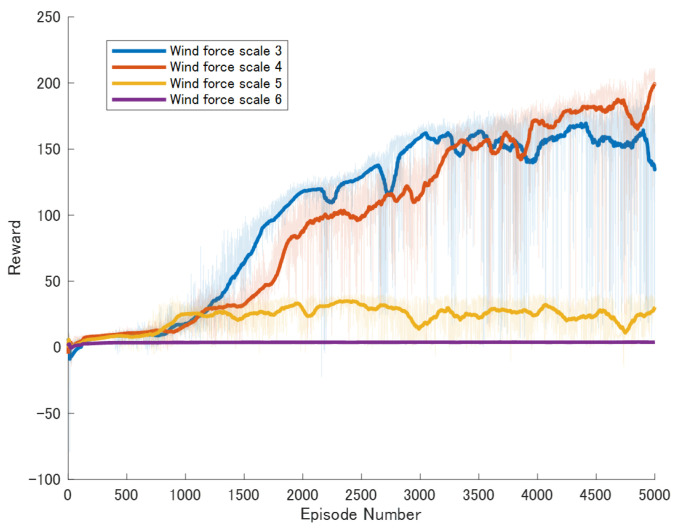
Result of An enhanced DDPG model featuring Reward Reshaping.

**Figure 8:**
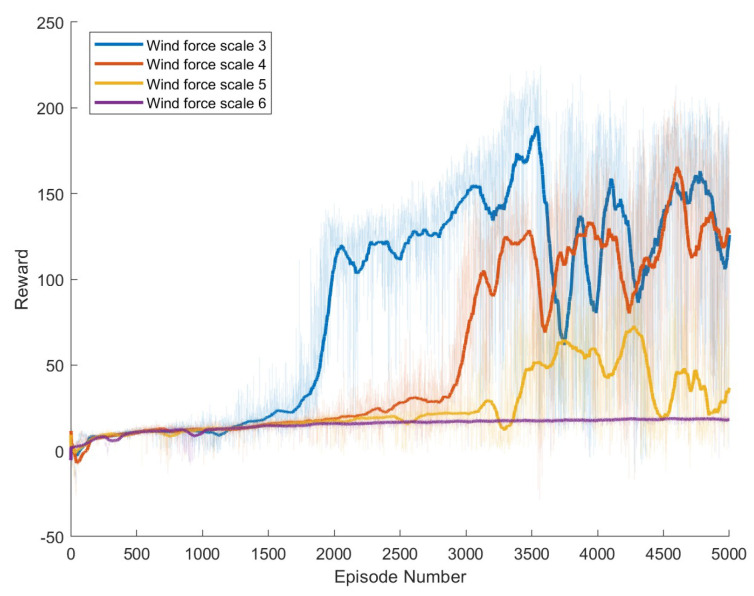
Result of DDPG model augmented with Adaptive Exploration and Hindsight Experience Replay.

**Figure 9:**
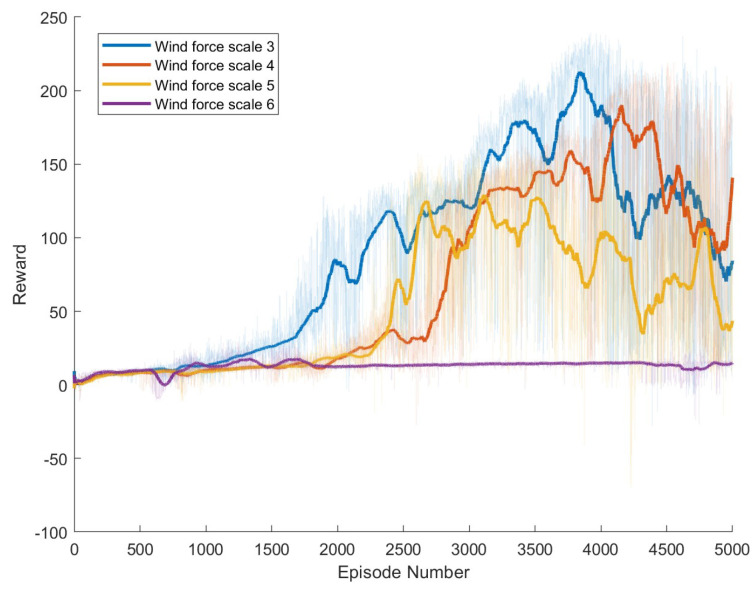
Result of The Proposed Method.

**Figure 10:**
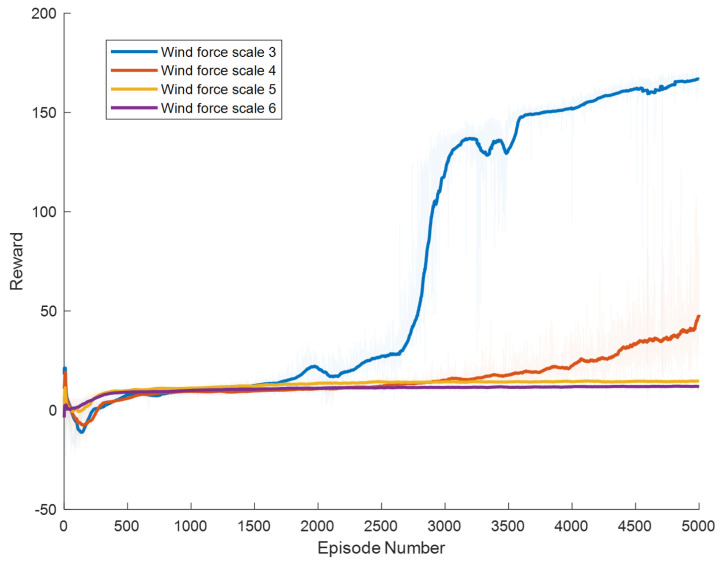
Result of The SAC Model.

**Figure 11:**
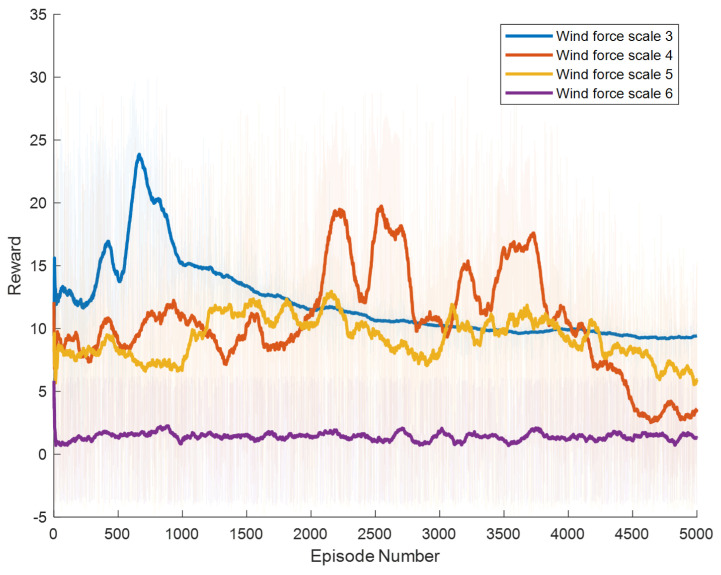
Result of The A3C Model.

Specific experimental results are displayed in Figure 6, Figure 7, Figure 8, Figure 9, Figure 10 and Figure 11, which depict the dynamic changes during the training process. The horizontal axis in these graphs represents the number of training rounds, while the vertical axis shows the average reward obtained by the agents in each episode. From these figures, it is observable that as training progresses, the agents gradually receive increasing rewards, indicating that the agents are progressively optimizing their adaptation to the environment through learning. Higher rewards suggest that the agents are more likely to take actions beneficial to their movement, effectively coping with the impact of wind speeds. By comparing the performance of SAC and A3C with our proposed method, we can determine which algorithm better adapts to the environmental challenges and achieves superior results in terms of reward optimization.

The SAC algorithm can achieve stable reward growth at wind level 3, but its convergence speed is slower compared to DDPG. At wind level 4, due to the slow convergence, the rewards, although showing an upward trend, remain at a relatively low level. The A3C algorithm, due to its relatively simple structure, cannot effectively converge in complex situations with large action spaces, resulting in overall poor performance. Compared to these two methods, our proposed model demonstrates better convergence speed under strong wind conditions.

**Figure 12:**
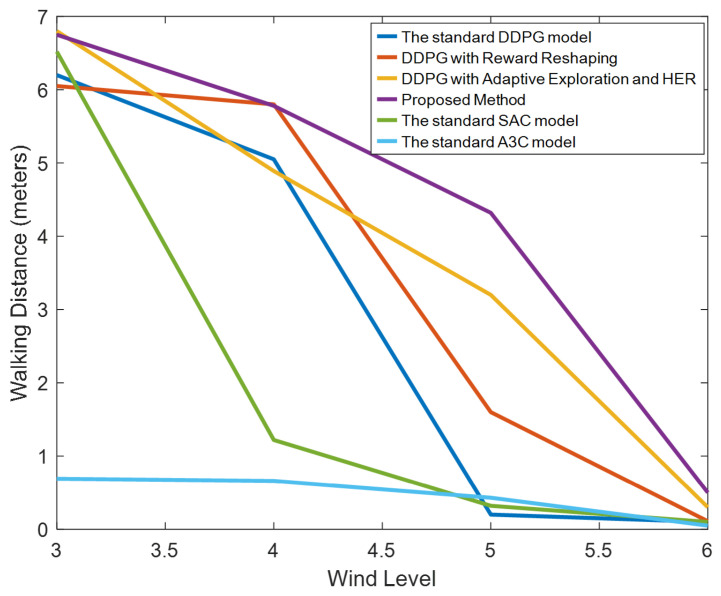
The distance a robot walked within ten seconds under four different methods. Methods A, B, C, and D correspond to the results in Figure 6, Figure 7, Figure 8 and Figure 9, tested under various wind conditions. From the experimental results, it is evident that our proposed method significantly outperforms the traditional DDPG algorithm and other methods under high wind speeds. Although the walking speed decreases at a wind speed of level 5, the robot can still walk normally.

Figure 12 illustrates the walking distances of a robot under various wind speeds. This figure presents four lines, each representing the distance traveled by different methods under varying wind levels within a span of 10 s. It is observed from the graph that, without employing HER and adaptive exploration, the performance remains relatively stable at wind levels 3 and 4. When the wind reaches level 5, robots controlled by standard DDPG immediately fall, whereas those utilizing reward reshaping can continue walking slowly without falling. Methods that implement HER and adaptive exploration, although restricted in walking distance, maintain stable movement under level 5 winds. At wind level 6, despite the robots falling immediately, they can learn to leap forward and dive as a strategy.

To demonstrate the superiority of adaptive exploration methods and HER in accelerating convergence, we analyzed the number of rounds required for the agents to reach a certain reward threshold during each experimental phase. In the experiments, it was determined that a reward value of 135 is indicative of the robot’s ability to achieve stable walking. Thus, we set this figure as a threshold for training. We compared the number of rounds it took for each method to first reach a reward of 135 in level 3 wind conditions. The outcomes of this comparison are presented in Table 3. It can be seen that the average number of episodes required to reach the standard reward value has decreased by 401.

## 4. Discussion

In conclusion, this study has developed a Deep Reinforcement Learning-based approach to enhance the stability and adaptability of biped robots under wind disturbances. A novel exploration strategy for robots navigating unknown environments was formulated, incorporating Reward Reshaping specifically tailored to practical scenarios. The effectiveness of this method has been empirically validated through simulations conducted across various wind speed conditions. The results demonstrate a significant improvement in the robots’ walking stability, especially in environments with wind speeds exceeding level 5, where performance notably surpasses that achieved using traditional reward functions.

The significance of this research extends beyond merely enhancing the practical utility of biped robots in complex environments; it also charts a new course for the future development of robotic technologies. As these technologies continue to mature, biped robots are poised to play a more critical role across various sectors, particularly in disaster response and outdoor exploration. Future research could delve into adaptive strategies for different environmental conditions and explore how these strategies can be applied to a broader array of robotic types to achieve wider application.

Furthermore, this study highlights the potential of integrating sophisticated sensor technologies and adaptive control algorithms to more effectively respond to dynamic environmental changes, such as sudden gusts of wind. By using the Ornstein–Uhlenbeck process to model wind velocity with temporal correlations, we also offer an innovative solution that makes robots explore more when the environment is severe, thus enhancing the robot’s performance in real-world walking scenarios.

Additionally, the incorporation of Hindsight Experience Replay and adaptive exploration contributes to overcoming the challenges associated with sparse rewards in reinforcement learning. This methodology not only speeds up the learning process but also ensures that robots can learn from unsuccessful attempts, turning failures into informative experiences that support rapid adaptation and optimization of walking strategies.

As we look forward, it is essential to conduct further investigations into the scalability of these methods and their applicability under varying operational conditions. Such research will help ensure that biped robots can reliably perform in a range of environments, thereby broadening their applications and further cementing their role in advancing robotic innovation and practical deployment in the field.

## 5. Conclusions

This study develops a Deep Reinforcement Learning-based method to improve the stability and adaptability of biped robots under windy conditions, employing a new exploration strategy and Reward Reshaping for practical scenarios. Through simulations, the approach showed enhanced stability in robots, especially in high-wind scenarios. The research also highlights the broader potential for advanced sensor technologies and adaptive control algorithms in dynamic environments, and it integrates techniques like Hindsight Experience Replay to expedite learning and optimize robot performance in real-world conditions. Looking ahead, further research is suggested to explore the scalability of these methods and their effectiveness in various operational settings, which could expand the practical uses and impact of biped robots in fields like disaster response and outdoor exploration.

## Figures and Tables

**Table 1:** Measurement of the Bipedal Robot.

Part	Measurement (mm)
Torso Height	80
Torso Length	80
Torso Width	50
Thigh Length	100
Shank Length	100
Foot Side Length	50

**Table 2:** Wind speed and average force change with change in wind level.

Wind Level	3	4	5	6
Wind Speed (m/s)	4.31	6.67	9.31	12.22
Average Force (N)	0.132	0.257	0.442	0.706

**Table 3:** Number of Episodes Required to Achieve 135 Reword by Different Methods. Since the A3C method do not achieve a reward of 135, it is not included.

Method	Episode Required
The standard SAC model	2890
The standard DDPG model	2578
DDPG with Reward Reshaping	2496
DDPG with Adaptive Exploration and HER	2004
Proposed Method	2269

## Data Availability

Data are contained within the article.

## Abbreviations

The following abbreviations are used in this manuscript:

DDPG	Deep Deterministic Policy Gradient
HER	Hindsight Experience Replay
OU	Ornstein–Uhlenbeck
DRL	Deep Reinforcement Learning
SAC	Soft Actor-Critic
MPC	Model Predictive Control
IMU	Inertial Measurement Unit
MLP	Multi Layer Perceptron
